# AsistIAM: Innovating the Acute Coronary Syndrome Approach in Primary Care Settings

**DOI:** 10.1002/clc.70313

**Published:** 2026-04-20

**Authors:** James S. Díaz, José L Añorga, Lucelly López, Cristian Vera, Henry Andrade, Johanna M. Vanegas

**Affiliations:** ^1^ Clínica Las Américas Auna Medellín Colombia; ^2^ Hospital General María Auxiliadora Lima Perú; ^3^ School of Health Sciences Universidad Pontificia Bolivariana Medellín Colombia; ^4^ School of Engineering Universidad Pontificia Bolivariana Medellín Colombia

**Keywords:** acute coronary syndrome, georeferencing, mHealth, mobile applications, primary care, reperfusion therapy, STEMI, ST‐segment elevation myocardial infarction

## Abstract

**Background:**

Early management of acute coronary syndrome (ACS) is critical. Clinical guidelines are more consistently implemented in high‐complexity hospitals; however, in rural and primary care settings multiple barriers limit timely diagnosis and treatment. This study aimed to develop and evaluate the usability of a mobile application designed to assist primary care physicians in the diagnostic approach and initial management of ACS.

**Methods:**

A mHealth tool named AsistIAM was developed with five modules: electrocardiogram pattern, reperfusion strategies, risk assessment, real‐time georeferencing, and educational content. Usability was assessed on a 1–7 scale under in both simulated and real‐world conditions. In the latter, a before‐and‐after study was performed with physicians from four rural primary healthcare centers and one referral hospital. In the pre‐implementation phase, participants completed a survey identifying barriers to ACS care. After using the app, they complete the same survey to analyze changes.

**Results:**

A total of 18 participants evaluated the app in simulated conditions and 59 in real‐world settings. Mean usability scores were high (6.52, in simulation and 6.18 in real‐world). The best‐rated items were ease of learning, interface satisfaction, and availability of functions. After app implementation, physicians reported improvements in ECG acquisition and interpretation (81.4%–100%), recognition of ST‐elevation patterns (25.6%–39.2%), identification of referral centers (88.1%–98.0%), and correct use of fibrinolytics (errors 44.1%–17.6%).

**Conclusions:**

AsistIAM represents an innovative tool to strengthen regional networks, improve ACS management and support timely clinical decision‐making. Usability results highlight its intuitive interface, functionality, and practical utility.

## Graphical abstract

AsistIAM, a mobile app for ACS management, was evaluated in Colombia. Usability testing showed high scores in simulated and real‐world conditions. The app enhanced ECG interpretation, referral identification, and fibrinolytic use, highlighting its usability, functionality, and potential to strengthen regional networks and support timely decision‐making.

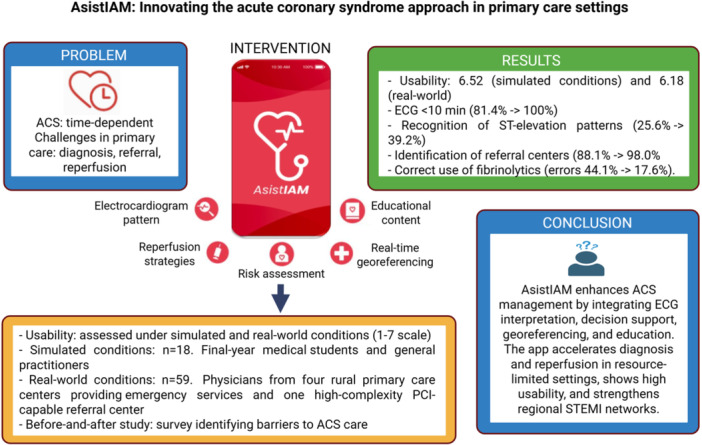

## 1 Introduction

Acute coronary syndromes (ACS) encompass conditions such as acute myocardial infarction (AMI) and unstable angina, representing the most severe manifestations of ischemic heart disease. Rapid recognition and early therapeutic action are critical determinants of patients outcomes [1]. The electrocardiogram (ECG) remains the first‐line diagnostic test and should be obtained and interpreted within 10 min of first medical contact [2]. However, in rural areas and primary care settings of low‐ and middle‐income countries, significant barriers such as limited healthcare resources, less experienced medical personnel, and geographic constraints delay the timely transfer of patients to specialized centers. Consequently, achieving guideline‐recommended timelines, particularly for ST‐segment elevation myocardial infarction (STEMI), is frequently no possible.

To address these challenges in rural areas and primary care settings, international guidelines recommend the creation of regional reperfusion networks, defined as coordinated systems of primary care centers, referral hospitals, and emergency services aimed at optimizing reperfusion strategies [3]. Mobile health (mHealth) technologies may enhance these networks by providing real‐time decision support, improving diagnosis accuracy, reducing delays, and facilitating education for healthcare providers [4, 5, 6].

In Colombia, ischemic heart disease is the leading cause of death and system delay is a known predictor of mortality in STEMI patients [7, 8, 9, 10]. With vast rural areas and unequal distribution of healthcare resources, innovative strategies are needed to bridge care gaps. In this context, we developed AsistIAM, a mobile application designed to support healthcare professionals in primary care by offering algorithm‐based guidance for ECG interpretation, reperfusion strategy selection, georeferencing of percutaneous coronary intervention (PCI) capable centers, risk stratification, and educational resources. This study reports the usability assessment of AsistIAM in simulated and real‐world conditions, focusing on its potential role in rural and primary care networks.

## 2 Material and Methods

### 2.1 Application Development

**Figure 1:**
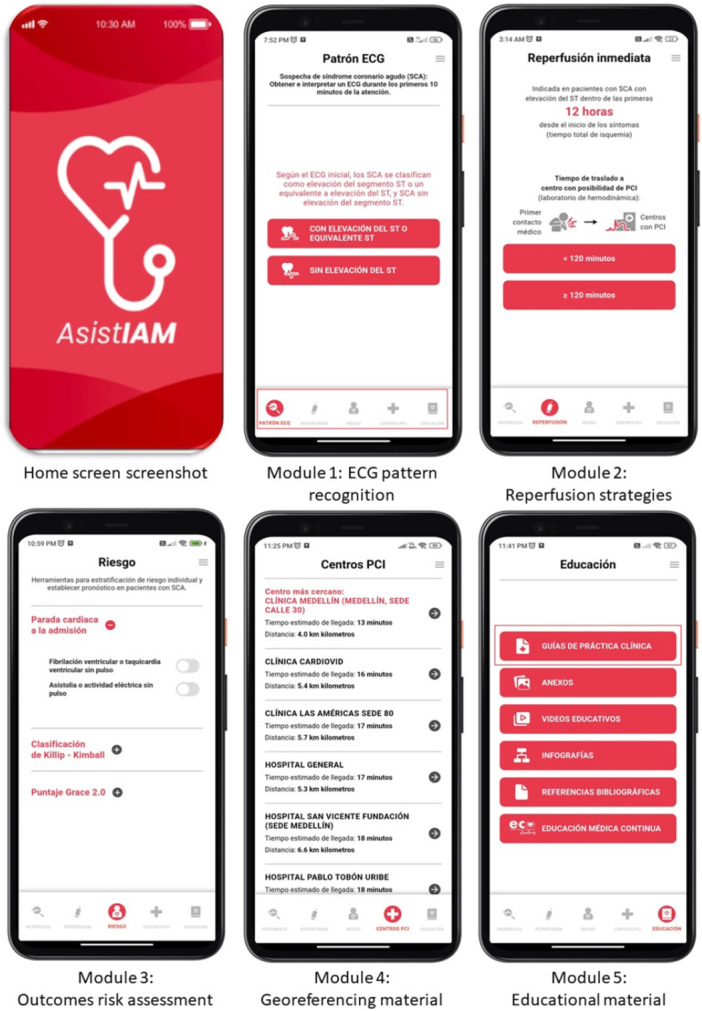
Screenshots of each module from AsistIAM.

AsistIAM was developed in Spanish following the Rapid Application Development (RAD) model [11]. Four phases guided the process: (1) requirements: analysis of ACS clinical guidelines and user needs; (2) interface design: iterative usability testing with clinicians; (3) construction: integration of validated medical algorithms; and (4) deployment: pilot implementation in both simulated and real‐world settings to evaluate the application's performance, usability, and acceptability among potential users. Technical details and a detailed explanation of each phase are provided in the supplementary material (Supporting Information S1: Figure 1).

The application included an intuitive interface comprising five key modules: (1) ECG pattern recognition; (2) Reperfusion strategies; (3) Risk assessment for adverse clinical outcome; (4) Georeferencing; and (5) Educational material (Figure 1). All algorithms and recommendations integrated into AsistIAM were based on clinical practice guidelines for ACS management (1):

1. ECG pattern recognition. This module enables rapid classification of ECG results based on the presence or absence of ST‐segment elevation, proving tailored clinical recommendations accordingly. It includes: ECG patterns interpretation, identification of the affected vascular territory for ST‐elevation patterns, determination of the need for immediate reperfusion, troponin levels interpretation, rule‐in and rule‐out algorithms, and concepts of myocardial injury and type 2 infarction for non‐ST‐elevation patterns.
2. Reperfusion strategies. AsistIAM suggest two main reperfusion options, depending on the estimated transfer time to a percutaneous coronary intervention (PCI) center: If the transfer time < 120 min, the app links to the georeferencing module to display the nearest PCI centers. If transfer time is ≥ 120 min, fibrinolysis is recommended as the initial reperfusion strategy, along with guidance on contraindications and potential complications of fibrinolytics therapy.
3. Risk Assessment. This module supports individualized risk stratification for ACS patients, helping to establish prognosis using three tools: cardiac arrest at admission, Killip‐Kimball classification [12], and GRACE score [13].
4. Georeferencing. This feature identifies the nearest PCI centers and integrates with Google Maps to calculate the fastest route, including distance and estimated travel time. This functionality is critical for facilitating rapid decision‐making in time‐sensitive ACS cases.
5. Educational material. The app provides comprehensive educational resources to enhance users´ clinical knowledge and technical skills. These include: Clinical practice guidelines, key reference materials developed for the app, educational videos, infographic, bibliographic references, and a Continuing Medical Education button linking to ECO Academy, a web‐based platform offering scientific resources to optimize AMI management [14].

### 2.2 Usability Under Simulated Conditions

To assess usability, three clinical cases of suspected ACS, each requiring distinct diagnostic and therapeutic decisions, were developed. Participants included final‐year medical students and general practitioners, representing the primary target users of the application. Additionally, three specialist (one in internal medicine and two in cardiology) provided expert input to ensure consistency with ACS management protocols. Eligibility criteria required access to a mobile device and an internet connection. After being informed about the research objectives and providing informed consent, participants downloaded the trial version of the application. Each participant first read the assigned clinical case and then used the app for approximately 20 min, exploring its different modules to assess whether, in a real scenario with a patient similar to the one described, the app could assist in diagnosis and initial management of ACS. Subsequently, usability was evaluated using the Mobile App Usability Questionnaire (MAUQ) and the items were rated on a 7‐point Likert scale (1 = strongly disagree, 7 = strongly agree) [15, 16]. Responses were collected anonymously through an online questionnaire. The simulated testing was designed not only to evaluate usability but also to guide app refinement. The survey instrument is provided in the supporting material.

### 2.3 Usability Testing in Real‐World Conditions

A before‐and‐after study was conducted in hospitals located in Antioquia, a large region of Colombia. Participants were physicians from four rural primary care centers providing emergency services and one high‐complexity PCI‐capable referral center in Medellín, the capital of Antioquia. Only physicians with access to a mobile device and internet connection were eligible. The characteristics of these centers are detailed in Supporting Information S1: Table 1. During the pre‐implementation phase, participants completed a baseline survey on diagnosis, treatment and barriers to ACS care. In the implementation phase, physicians were instructed to use the application with any patient presenting with suspected ACS during the study period, allowing adaptation and flexibility to each site's workflow. Subsequently, the application was used during 1 month in primary care centers and 2 weeks in the referral center (due to higher patient volume). In each hospital, a member of the research team was responsible for providing weekly reminders to encourage the use of the app and for addressing any questions or difficulties that arose regarding its operation. Finally, in the post‐implementation phase, participants repeated the baseline survey and completed the MAUQ with items rated on a 7‐point Likert scale (1 = strongly disagree, 7 = strongly agree) [15, 16].

### 2.4 Variables and Data Collection

Collected variables included sex, age, occupation, years of mobile device usage, years of app usage, and length of employment at the healthcare center. For ACS diagnosis and treatment, as well as the identification of related barriers, the study assessed: time required for ECG acquisition and interpretation, ability to differentiate ST‐segment elevation from non‐ST‐segment elevation patterns, timeliness for coronary reperfusion therapy, identification of nearby referral centers, barriers to transfer (administrative, geographic, economic), and use of thrombolytics, antiplatelets, statins, and anticoagulants. The online questionnaires incorporate validation mechanism including predefined values, duplicate‐entry restrictions, and field deactivation mechanisms to ensure data integrity. An open‐ended section allowed participants to provide qualitative feedback on their experience. All variables were self‐reported and not verified against institutional records.

### 2.5 Statistical Analysis

Qualitative variables were summarized using absolute frequencies, while continuous quantitative variables where assessment for normality using the Shapiro‐Francia test. Since these variables did not follow a normal distribution, they were reported using the median and interquartile range (IQR). MAUQ scores were reported as means and standard deviations. All statistical analyses were conducted using Stata V.18.

### 2.6 Ethical Considerations

This study adhered to international ethical standards, including the Nuremberg Code, the Declaration of Helsinki, the Belmont Report, the World Health Organization's Good Clinical Practice guidelines, and the CIOMS 2009 Guidelines for Epidemiological Studies. The research protocol received ethical approval from the Ethics Committees of Pontifical Bolivarian University (Approval No. 09, 2022) and Clínica Las Américas Auna (Approval No. 220, 2024). Informed consent was obtained from all participants.

## 3 Results

### 3.1 Usability in Simulated Conditions

The trial version of AsistIAM was evaluated using the MAUQ among 18 participants, including 12 medical students, three general practitioners, one internal medicine specialist, and two cardiology subspecialists. The sex distribution was balanced, with a median age of 24 years (IQR: 24–34). Participants reported a median of 14.5 years (IQR: 10–19) using mobile devices and 10 years (IQR: 10–14) using mobile applications (Table 1).

**Table 1:** Demographic characteristics and mobile device usage of the study population.

Characteristics	Simulated conditions *n* = 18	Real‐world conditions *n* = 59
Sex, *n*		
Male	9	35
Female	9	24
Occupation, *n*		
Medical student	12	0
General practitioner	3	58
Specialist	3	1
Age		
Median (Interquartile range)	24 (24–34)	29 (26–34)
Years of mobile device use		1
Median (Interquartile range)	14.5 (10–19)	15 (12–15)
Years of mobile application use		
Median (Interquartile range)	10 (10–14)	10 (10–15)

The mean overall usability score was 6.52 (SD: 0.34). Subspecialists and specialists reported the lowest scores (mean 6.30; SD: 0.76) compared to medical students (mean 6.52; SD: 0.40) and general practitioners (mean 6.79; SD: 0.11). Among the 21 usability aspects evaluated, the highest‐rated items were comfort in using AsistIAM in social settings and the likelihood of future use, both scoring 6.94 (SD: 0.24). In contrast, the lowest‐rated aspects were ease and speed of error recovery (5.94; SD: 1.35) and availability to required functionalities (5.89; SD: 1.28) (Table 2).

**Table 2:** Scores for each question included in the usability questionnaire.

Statements	Simulated conditions *n* = 18			Real‐world conditions *n* = 46		
	Mean	SD	Min ‐ Max	Mean	SD	Min ‐ Max
**Ease of use and satisfaction**						
1. The app was easy to use	6.55	0.70	5–7	6.37	0.83	4–7
2. It was easy for me to learn to use the app	6.67	0.59	5–7	6.46	0.69	4–7
3. I like the interface of the app	6.55	0.78	5–7	6.43	0.66	4–7
4. The information in the app was well organized, so I could easily find the information I needed	6.22	0.94	4–7	6.41	0.75	4–7
5. I feel comfortable using this app in social settings	6.94	0.24	6–7	6.33	0.97	3–7
6. The amount of time involved in using this app has been fitting for me	6.61	0.85	4–7	6.20	1.15	1–7
7. I would use this app again	6.94	0.24	6–7	6.26	1.18	1–7
8. Overall, I am satisfied with this app	6.33	0.91	4–7	6.35	0.87	3–7
**System information arrangement**						
9. Whenever I made a mistake using the app, I could recover easily and quickly	5.94	1.35	3–7	6.26	0.85	4–7
10. This mHealth app provides an acceptable way to deliver healthcare services	6.72	0.46	6–7	6.41	0.75	4–7
11. The app adequately acknowledged and provided information to let me know the progress of my action	6.61	0.78	4–7	6.20	1.13	1–7
12. The navigation was consistent when moving between screens	6.72	0.57	5‐7	6.33	0.82	4–7
13. The interface of the app allowed me to use all the functions offered by the app	6.67	0.77	4–7	6.43	0.65	4–7
14. This app has all the functions and capabilities I expected it to have	5.89	1.28	2–7	6.04	1.15	2–7
**Usefulness**						
15. The app would be useful for my healthcare practice	6.83	0.38	6–7	6.28	1.11	1–7
16. The app improved my access to delivering healthcare services	6.61	1.04	3–7	5.87	1.31	1–7
17. The app helped me manage my patients’ health effectively	6.78	0.55	5–7	6.00	1.32	1–7
18. The app made it convenient for me to communicate with my patients	6.39	0.92	4–7	5.83	1.32	1–7
19. Using the app, I had many more opportunities to interact with my patients	6.00	1.53	1–7	5.70	1.41	1–7
20. I felt confident that any information I sent to my patients using the app would be received	6.50	0.86	5–7	5.83	1.39	1–7
21. I felt comfortable communicating with my patients using the app	6.55	0.92	4–7	5.74	1.40	1–7
Total	6.52	0.34		6.18	0.36	

Abbreviation: SD, Standard deviation.

Based on the feedback, a revised version of AsistIAM was developed and subsequently tested under real‐world conditions. This version included: Reminders to reinforce clinical decision‐making, expanded audiovisual educational materials, refined diagnostic algorithms to encompass all ACS presentations, including non‐ST‐segment elevation myocardial infarction (NSTEMI) and unstable angina, integration of high‐sensitivity troponin testing algorithms, incorporating rule‐in and rule‐out pathways for myocardial infarction, and inclusion of myocardial injury and type 2 MI concepts, alongside risk stratification tools.

### 3.2 Usability in Real‐World Settings

A total of 59 physicians participated in the five hospitals, with 39 (66.1%) being male and a median age of 29 years (IQR: 26–34). The majority were general practitioners (*n* = 58), with only one emergency medicine specialist (Table 1).

For the usability assessment, 46 physicians were included, as 11 encountered issues downloading the app on Android devices, and two physicians left their positions during the study period. The mean usability score was 6.18 (SD: 0.36). The highest‐rated aspects were: Ease of learning to use the app (mean 6.46, SD: 0.69), satisfaction with the interface (mean 6.43, SD: 0.66), and available of core functions (mean 6.43, SD: 0.65). Conversely, the lowest‐rated aspects were: Comfort in communicating with patients using the AsistIAM (mean 5.74, SD: 1.40) and perceived increase in patient interaction opportunities (mean 5.70, SD: 1.41) (Table 2).

Following the implementation of AsistIAM, there was a notable improvement in self‐reported ECG acquisition and interpretation within the first 10 min of patient care (before: 81.4%, after: 100%). Additional improvements included: Recognition of ST‐segment elevation patterns (“always” category: before 25.6% vs. after 39.2%), identification of nearby PCI referral centers (before 88.1% vs. after 98.0%), reduction in errors related to troponin levels interpretation (before 47.5% vs. after 37.2%), and a decrease in lack of knowledge regarding usage and dosage of fibrinolytic drugs (before 44.1% vs. after 17.6%) (Table 3).

**Table 3:** Comparison of factors related to the diagnosis and treatment of acute coronary syndrome (ACS) before and after implementation of the application in real‐world conditions.

Factor	Implementation	
	Before *n* = 59 (%)	After *n* = 51 (%)
Hospital		
A	16 (27.1)	NA
B	9 (15.3)	NA
C	12 (20.3)	NA
D	6 (10.2)	NA
E	16 (27.1)	NA
Time at the institution		
Less than 6 months	20 (33.9)	NA
Between 6 months and 1 year	11 (18.6)	NA
Between 1 and 5 years	25 (42.4)	NA
More than 5 years	3 (5.1)	NA
Self‐reported ECG acquisition and interpretation within the first 10 min of care		
No	1 (1.7)	0
Yes	48 (81.4)	51 (100)
Only acquisition	10 (17.0)	0
Difference between ACS with and without ST‐segment elevation		
Sometimes	4 (6.8)	4 (7.8)
Almost always	34 (57.6)	27 (52.9)
Always	21 (25.6)	20 (39.2)
Timely coronary reperfusion treatment		
Never	5 (8.5)	0
Almost never	0	5 (9.8)
Sometimes	15 (25.4)	11 (21.6)
Almost always	30 (50.9)	28 (54.9)
Always	9 (15.2)	7 (13.7)
Identification of nearby referral centers		
No	7 (11.9)	1 (2.0)
Yes	52 (88.1)	50 (98.0)
Challenges in the diagnosis and management of ACS		
Interpretation of symptoms	10 (17.0)	10 (19.6)
ECG interpretation	33 (55.9)	25 (49.0)
Troponin interpretation	28 (47.5)	19 (37.2)
Administrative barriers to referral	34 (57.6)	28 (54.9)
Lack of transportation for referral	14 (23.7)	10 (19.6)
Geographic barriers to referral	23 (39.0)	17 (33.3)
Economic barriers to referral	20 (33.9)	14 (27.5)
Challenges in the use of fibrinolytics		
Lack of knowledge about usage and dosage	26 (44.1)	9 (17.6)
Lack of knowledge about contraindications and adverse effects	25 (42.4)	12 (23.5)
Fear of using them	29 (49.2)	14 (27.5)
Not available at the institution	44 (74.6)	41 (80.4)
Availability of antiplatelets, statins, anticoagulants	42 (71.2)	41 (80.4)

Abbreviations: ECG, Electrocardiogram; NA, Not applicable.

## 4 Discussion

Cardiovascular diseases (CVD) remain the leading cause of mortality worldwide, accounting for nearly one‐third of all global deaths, with ischemic heart disease as the primary contributor to premature mortality [17]. More than 75% of CVD‐related deaths occur in low‐ and middle‐income countries, where access timely diagnosis and reperfusion therapies for ACS remains limited [18, 19]. Although global mortality rates from CVD are declining, this reduction is occurring more slowly in resource‐constrained regions, underscoring the urgent need for context‐specific strategies to improve ACS management [17]. For STEMI, in particular, is recommended the establishment of regional reperfusion networks to reduce delays and optimize outcomes [20, 21, 22, 23, 24]. However, the implementation of such networks in countries with heterogeneous health systems, such as Colombia, faces significant logistical, geographic, and infrastructural challenges. In this context, mobile health (mHealth) tools emerge as potentially valuable interventions to standardize care processes and support clinical decision‐making at the primary care level.

This study reports the development and usability evaluation of AsistIAM, a mHealth application designed to support physicians in rural and primary care settings in a region of Colombia. The app integrates algorithm‐based decision support for ECG interpretation with georeferencing capabilities that suggest the most appropriate reperfusion strategy, considering PCI availability and transfer times. Additionally, it incorporates educational modules with updated clinical practice guidelines, videos, and infographics to strengthen continuous medical education. ECG interpretation is a critical step in ACS diagnosis, yet previous studies have reported substantial discrepancies between emergency physicians and cardiologists in interpreting ECG [25, 26, 27]. These discrepancies are even more pronounced among junior doctors where physicians in training showed lower concordance with specialist interpretations [28]. By addressing two critical challenges in ACS management: diagnostic uncertainty and delayed decision‐making, AsistIAM provides both practical and educational value to frontline physicians.

The usability evaluation of AsistIAM yielded encouraging results. Physicians reported high overall usability scores, averaging 6.52 under simulated conditions and 6.18 in real‐world settings, as measured by the MAUQ. The most valued aspects included ease of learning, intuitive interface, and likelihood of continued use. Importantly, physicians in training highlighted an improvement in their ability to recognize ST‐segment elevation patterns after using the application, reinforcing its potential educational benefit. Conversely, the lowest‐rated features were related to error recovery and communication with patients through the app, limitations consistent with its design as a decision‐support tool for professionals rather than a platform for patient interaction. Based on early feedback, the app was refined to include enhanced audiovisual educational content, updated algorithms covering NSTEMI and unstable angina, integration of high‐sensitivity troponin pathways, and risk stratification tools. These iterative improvements suggest that AsistIAM can evolve into a comprehensive aid for the diagnosis and initial management of the full spectrum of ACS.

Previous studies have reported on mobile applications for ACS, mainly focused on improving AMI treatment workflows. For example, in 2016, Studencan et al. (Slovakia) introduced an app that allowed prehospital personnel to transmit ECG and communicate directly with on‐call cardiologists at PCI centers, reducing ischemic times and preventing unnecessary transfers for non‐AMI cases [29]. In the United States, Dickson et al. developed STOP STEMI, which enabled physicians and on‐call teams to share clinical data and ECGs in hospitals with catheterization labs, resulting in a 22% reduction in door‐to‐balloon time [30]. To our knowledge, no applications similar to AsistIAM have been developed in Colombia or Latin America, highlighting the novelty and potential regional impact of this initiative.

The findings of this study also suggest that AsistIAM could serve as a catalyst for the creation of a regional ACS care network. By providing real‐time guidance at the first point of contact, particularly in rural and primary care centers without PCI capability, the app could reduce variability in early treatment and streamline referral processes. The georeferencing feature supports timely reperfusion decisions, aligning with international guideline recommendations for regionalized STEMI care. If scaled, AsistIAM could help build a more coordinated system in which primary care physicians and referral centers collaborate more efficiently to improve outcomes.

Significant barriers remain to the establishment of such networks. These include limited availability of advanced diagnostic tools, variability in ambulance and transfer logistics, unequal distribution of PCI centers, and systemic issues such as fragmented health insurance schemes. While AsistIAM can directly address barriers related to physician knowledge, ECG interpretation, and initial decision‐making, it cannot resolve structural limitations such as transport delays, inter‐institutional coordination, or inadequate resources. Therefore, although the results of this usability assessment are likely generalizable to similar rural and resource‐limited settings, successful implementation in other health systems may require adaptation to local infrastructure, referral pathways, and governance structures. Ultimately, scaling a regional ACS network will depend not only on digital innovations such as AsistIAM but also on broader investments tailored to the specific context of each healthcare environment.

This study has some limitations. The sample size was relatively small, which may limit the generalizability of findings. Nonetheless, small‐scale usability testing is a widely accepted approach for refining app design and functionality before broader deployment, ensuring that critical issues are addressed prior to full‐scale implementation. On the other hand, the variables measured were self‐reported and metrics as frequency of use, educational module completion or acquisition of EKG were not established nor measured by the sites themselves. However, in each hospital, a member of the research team conducted ongoing follow‐up to remind physicians about the use of the app and to address any questions, in order to maximize its use and ensure that the results could be attributed to the application itself rather than merely to the awareness of being part of a study. Beyond usability, further studies will be needed to evaluate key implementation outcomes, including adoption, sustained use, fidelity and incorporate objective site‐level data in order to ensure long‐term integration into diverse healthcare settings.

## 5 Conclusions

AsistIAM illustrates the potential of mHealth technology to improve the diagnosis and management of ACS. By combining ECG interpretation, algorithm‐based decision support, real‐time georeferencing, and an educational module, the app supports rapid decision‐making, particularly in resource‐limited settings. Usability testing confirmed its user‐friendly design and functionality, underscoring its value as both a clinical support and educational tool. Moreover, AsistIAM offers a novel approach that could strengthen the development and integration of regional STEMI networks.

## Ethics Statement

This study adhered to international ethical standards, including the Nuremberg Code, the Declaration of Helsinki, the Belmont Report, the World Health Organization's Good Clinical Practice guidelines, and the CIOMS 2009 Guidelines for Epidemiological Studies. The research protocol received ethical approval from the Health Research Ethics Committee of Pontifical Bolivarian University (Approval No. 09, 2022) and the Research Ethics Committee at Clínica Las Américas Auna (Approval No. 220, 2024).

## Consent

Informed consent was obtained from all participants prior to their involvement in the study.

## Conflicts of Interest

The authors declare no conflicts of interest.

## Supporting information

Supporting file 1

Supporting file 2

## Data Availability

Data available on request from the authors.
