# Supplementary material for: AsistIAM: Innovating the Acute Coronary Syndrome Approach in Primary Care Settings
Source: Clin Cardiol. 2026 Apr 20;49(4):e70313. doi: 10.1002/clc.70313 (PMC13094408; doi:10.1002/clc.70313)
Supplement: Supplementary file 1 — Supporting file 1 [file CLC-49-e70313-s001.docx]

**SUPPLEMENTARY MATERIAL**

**Application development**

The mobile application was developed using Flutter, a cross-platform framework written in Dart, which enables rapid prototyping, high-performance user interface rendering, and maintainable code. Firebase Core and Cloud Firestore provide real-time data synchronization and secure cloud storage, while the Geolocator and Google Maps Flutter libraries support precise location tracking and mapping capabilities. State management is handled through Riverpod for efficient data sharing, while go_router facilitates flexible, declarative navigation. Additionally, libraries such as pdf, printing, and video player enhance multimedia interaction and document handling. Collectively, these technologies contribute to the app’s scalability, reliability, and overall user experience, making them particularly beneficial for the project’s research focus on data collection, analysis, and real-time information delivery.

To accelerate prototyping and user feedback while minimizing prolonged development and testing cycles, we employed the Rapid Application Development (RAD) model (11). The development process followed four key phases: (1) Requirements (Planning): Systematic analysis of ACS clinical guidelines and user needs, defining core modules such as ECG recognition, reperfusion strategies, georeferencing, and educational materials; (2) User interface design: Iterative usability testing with clinicians to refine screen layouts and navigation flows; (3) Construction: Integration of validated medical content and system modules through multiple coding iterations and prototype evaluations; and (4) Cutover (Deployment): Implementation in both simulated and real-world conditions to evaluate the application’s performance, usability, and acceptability among potential users. A detailed explanation of each phase is provided in **Figure S1**

| 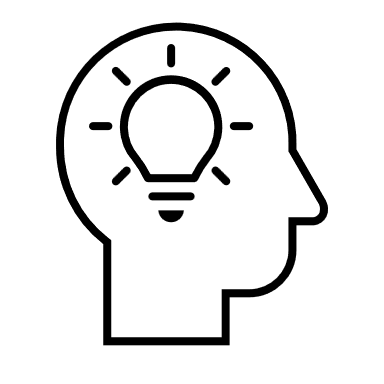PHASE 1 | Requirements (planning): Compilation of existing medical knowledge on the topic, such as clinical practice guidelines and articles in indexed journals. |
| --- | --- |
| 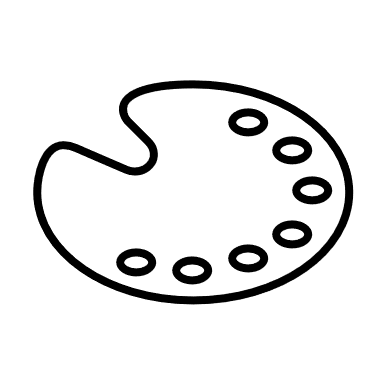PHASE 2 | User interface design: Selection of the app’s style, format, logo, and navigation interaction. |
| 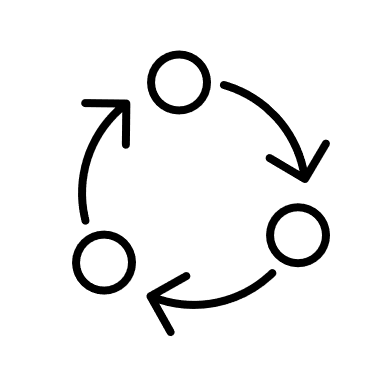PHASE 3 | Construction: Application programming and coding. |
| 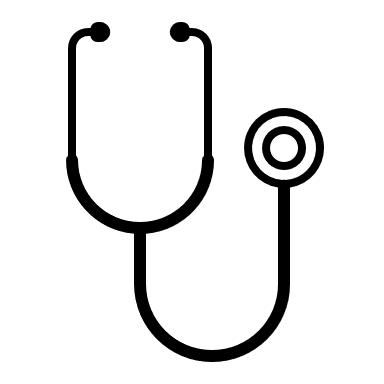PHASE 4 | Cutover: Evaluation under simulated and real-world conditions. |

**Figure S1.** Development phases of mobile applications using the Rapid Application Development (RAD) model

**Phase 1: Requirements and planning**

A comprehensive review of published articles from indexed scientific journals and Clinical Practice Guidelines (CPGs) from the European Society of Cardiology and the American College of Cardiology was conducted. Key information related to electrocardiographic findings and management algorithms was extracted to determine the most appropriate reperfusion strategy based on the time elapsed since symptom onset (fibrinolysis vs. percutaneous coronary intervention [PCI]). Additionally, the geographic locations of the nearest reference centers with hemodynamic services were identified for the georeferencing process. For fibrinolysis, scientific data on available treatment options, including drug selection, dosages, administration methods, and contraindications, were compiled. Furthermore, strategies for patient monitoring, identification of warning signs, and adjunctive treatments such as anticoagulation and antiplatelet therapy were documented to support initial patient management. To standardize the application's functionalities, use cases were defined using Unified Modeling Language (UML). Class and sequence diagrams were also developed to structure the application’s architecture. Regular meetings with clinical and technical teams were held to refine the system’s design based on expert feedback. Finally, a content framework for the application was outlined, specifying the types of media (video, audio, text, etc.) required for the development of audiovisual educational materials.

**Phase 2: User interface design**

Mockups were created to provide a realistic representation of the mobile application’s prototype, illustrating key user interface components and essential functionalities. A structured data model was developed alongside an information architecture designed in XMind, allowing step-by-step visualization of the application’s interface and its interactions. This architecture incorporated hyperlinks to foundational algorithms and reperfusion strategies to facilitate navigation. A graphical user interface (GUI) was designed using Adobe Illustrator, accompanied by a flow diagram to visualize the relationships between different modules. Additionally, educational content was integrated into the application, including video lectures on electrocardiogram interpretation and reperfusion strategies.

**Phase 3: Development and implementation**

The initial prototype was developed with the core functionalities outlined in the previous phases. Flutter, an open-source framework developed by Google, was chosen for cross-platform development on iOS and Android. The CLEAN architecture (Component Layered Architecture for N-tier) was implemented to ensure modularity, separation of concerns, and scalability of the system. The development process involved iterative refinements until a stable version of the application was achieved and ready for user testing. Georeferencing features were incorporated to identify care centers with hemodynamic services for PCI, enabling real-time estimation of distance and travel time to the nearest PCI-capable center via Google Maps. Additionally, an educational infographic was created and integrated into the app’s workflow through interactive hyperlinks.

**Phase 4: Usability testing and evaluation**

A usability assessment was conducted in both simulated and real-world conditions to evaluate the application’s performance, usability, and acceptability among potential users, including medical students, general practitioners, and specialists. The methodology used for this evaluation is described in detail within the manuscript.

**Table S1.** Characteristics of hospitals where usability assessment was conducted under real-world conditions

| **Characteristic** | **Hospital A** | **Hospital B** | | **Hospital C** | **Hospital D** | **Hospital E** |
| --- | --- | --- | --- | --- | --- | --- |
| **Level of complexity** | Primary care | Primary care | Primary care | | Primary care | High complexity |
| **Estimated monthly ACS patient count** | 4 | 3 | 3 | | 2 | 100 |
| **Estimated emergency department physician count** | 14 | 8 | 12 | | 6 | 26 |
| **Emergency department physician** | General practitioners | General practitioners | General practitioners | | General practitioners | General and emergency medicine practitioners |
| **Availability of ECG 24 hours per day** | Yes | Yes | Yes | | Yes | Yes |
| **Availability of fibrinolytic** | No | No | No | | No | Yes |
| **Availability of 24-hour ambulance service for patient referral** | Yes | Yes | Yes | | Yes | Yes |
| **Availability of troponin** | Yes, conventional | No | Yes, conventional | | No | Yes, high sensitivity |
