# Supplementary material for: AsistIAM: Innovating the Acute Coronary Syndrome Approach in Primary Care Settings
Source: Clin Cardiol. 2026 Apr 20;49(4):e70313. doi: 10.1002/clc.70313 (PMC13094408; doi:10.1002/clc.70313)
Supplement: Supplementary file 2 — Supporting file 2 [file CLC-49-e70313-s002.pdf]

## Funcionalidad de la aplicación

Cada pregunta de esta sección tiene 7 opciones de respuesta de la siguiente manera:
1 - Totalmente en desacuerdo
2 - En desacuerdo
3 - Algo en desacuerdo
4 - Ni de acuerdo ni en desacuerdo
5 - Algo de acuerdo
6 - De acuerdo
7 - Totalmente de acuerdo.

8. ¿La aplicación fue fácil de usar? *

| | 1. Totalmente en desacuerdo | 2. En desacuerdo | 3. Algo en desacuerdo | 5. Ni de acuerdo ni en desacuerdo | 5. Algo de acuerdo | 6. De acuerdo | 7. Totalmente de acuerdo |
|---|---|---|---|---|---|---|---|
| | ○ | ○ | ○ | ○ | ○ | ○ | ○ |

9. Fue fácil para mí aprender a usar la aplicación *

| | 1. Totalmente en desacuerdo | 2. En desacuerdo | 3. Algo en desacuerdo | 5. Ni de acuerdo ni en desacuerdo | 5. Algo de acuerdo | 6. De acuerdo | 7. Totalmente de acuerdo |
|---|---|---|---|---|---|---|---|
| | ○ | ○ | ○ | ○ | ○ | ○ | ○ |

10. Me gusta la interfaz de la aplicación *

| | 1. Totalmente en desacuerdo | 2. En desacuerdo | 3. Algo en desacuerdo | 5. Ni de acuerdo ni en desacuerdo | 5. Algo de acuerdo | 6. De acuerdo | 7. Totalmente de acuerdo |
|---|---|---|---|---|---|---|---|
| | ○ | ○ | ○ | ○ | ○ | ○ | ○ |

11. La información en la aplicación estaba bien organizada, así que pude encontrar fácilmente la información que necesitaba *

| 1. Totalmente en desacuerdo | 2. En desacuerdo | 3. Algo en desacuerdo | 5. Ni de acuerdo ni en desacuerdo | 5. Algo de acuerdo | 6. De acuerdo | 7. Totalmente de acuerdo |
|---|---|---|---|---|---|---|
| ○ | ○ | ○ | ○ | ○ | ○ | ○ |

12. Me siento cómodo usando esta aplicación en entornos sociales *

| 1. Totalmente en desacuerdo | 2. En desacuerdo | 3. Algo en desacuerdo | 5. Ni de acuerdo ni en desacuerdo | 5. Algo de acuerdo | 6. De acuerdo | 7. Totalmente de acuerdo |
|---|---|---|---|---|---|---|
| ○ | ○ | ○ | ○ | ○ | ○ | ○ |

13. La cantidad de tiempo invertido en usar esta aplicación ha sido adecuada para mí *

| 1. Totalmente en desacuerdo | 2. En desacuerdo | 3. Algo en desacuerdo | 5. Ni de acuerdo ni en desacuerdo | 5. Algo de acuerdo | 6. De acuerdo | 7. Totalmente de acuerdo |
|---|---|---|---|---|---|---|
| ○ | ○ | ○ | ○ | ○ | ○ | ○ |

14. Volvería a usar esta aplicación *

| 1. Totalmente en desacuerdo | 2. En desacuerdo | 3. Algo en desacuerdo | 5. Ni de acuerdo ni en desacuerdo | 5. Algo de acuerdo | 6. De acuerdo | 7. Totalmente de acuerdo |
|---|---|---|---|---|---|---|
| ○ | ○ | ○ | ○ | ○ | ○ | ○ |

15. En general, estoy satisfecho con esta aplicación *

| | 1. Totalmente en desacuerdo | 2. En desacuerdo | 3. Algo en desacuerdo | 5. Ni de acuerdo ni en desacuerdo | 5. Algo de acuerdo | 6. De acuerdo | 7. Totalmente de acuerdo |
|---|---|---|---|---|---|---|---|
| | ◯ | ◯ | ◯ | ◯ | ◯ | ◯ | ◯ |

16. Siempre que cometía un error usando la aplicación, podía recuperarme fácil y rápidamente *

| | 1. Totalmente en desacuerdo | 2. En desacuerdo | 3. Algo en desacuerdo | 5. Ni de acuerdo ni en desacuerdo | 5. Algo de acuerdo | 6. De acuerdo | 7. Totalmente de acuerdo |
|---|---|---|---|---|---|---|---|
| | ◯ | ◯ | ◯ | ◯ | ◯ | ◯ | ◯ |

17. La aplicación proporciona una forma aceptable de ofrecer servicios de salud *

| | 1. Totalmente en desacuerdo | 2. En desacuerdo | 3. Algo en desacuerdo | 5. Ni de acuerdo ni en desacuerdo | 5. Algo de acuerdo | 6. De acuerdo | 7. Totalmente de acuerdo |
|---|---|---|---|---|---|---|---|
| | ◯ | ◯ | ◯ | ◯ | ◯ | ◯ | ◯ |

18. La aplicación reconocía adecuadamente y proporcionaba información para que yo conociera el progreso de mis acciones *

| | 1. Totalmente en desacuerdo | 2. En desacuerdo | 3. Algo en desacuerdo | 5. Ni de acuerdo ni en desacuerdo | 5. Algo de acuerdo | 6. De acuerdo | 7. Totalmente de acuerdo |
|---|---|---|---|---|---|---|---|
| | ◯ | ◯ | ◯ | ◯ | ◯ | ◯ | ◯ |

19. La navegación era consistente al moverme entre pantallas *

| 1. Totalmente en desacuerdo | 2. En desacuerdo | 3. Algo en desacuerdo | 5. Ni de acuerdo ni en desacuerdo | 5. Algo de acuerdo | 6. De acuerdo | 7. Totalmente de acuerdo |
|---|---|---|---|---|---|---|
| ○ | ○ | ○ | ○ | ○ | ○ | ○ |

20. La interfaz de la aplicación me permitió usar todas las funciones que ofrecía la aplicación *

| 1. Totalmente en desacuerdo | 2. En desacuerdo | 3. Algo en desacuerdo | 5. Ni de acuerdo ni en desacuerdo | 5. Algo de acuerdo | 6. De acuerdo | 7. Totalmente de acuerdo |
|---|---|---|---|---|---|---|
| ○ | ○ | ○ | ○ | ○ | ○ | ○ |

21. Esta aplicación tiene todas las funciones y capacidades que esperaba que tuviera *

| 1. Totalmente en desacuerdo | 2. En desacuerdo | 3. Algo en desacuerdo | 5. Ni de acuerdo ni en desacuerdo | 5. Algo de acuerdo | 6. De acuerdo | 7. Totalmente de acuerdo |
|---|---|---|---|---|---|---|
| ○ | ○ | ○ | ○ | ○ | ○ | ○ |

22. La aplicación sería útil para mi práctica de atención médica *

| 1. Totalmente en desacuerdo | 2. En desacuerdo | 3. Algo en desacuerdo | 5. Ni de acuerdo ni en desacuerdo | 5. Algo de acuerdo | 6. De acuerdo | 7. Totalmente de acuerdo |
|---|---|---|---|---|---|---|
| ○ | ○ | ○ | ○ | ○ | ○ | ○ |

23. La aplicación mejoró mi acceso a la prestación de servicios de salud *

| | 1. Totalmente en desacuerdo | 2. En desacuerdo | 3. Algo en desacuerdo | 5. Ni de acuerdo ni en desacuerdo | 5. Algo de acuerdo | 6. De acuerdo | 7. Totalmente de acuerdo |
|---|---|---|---|---|---|---|---|
| | ◯ | ◯ | ◯ | ◯ | ◯ | ◯ | ◯ |

24. La aplicación me ayudó a gestionar eficazmente la salud de mis pacientes *

| | 1. Totalmente en desacuerdo | 2. En desacuerdo | 3. Algo en desacuerdo | 5. Ni de acuerdo ni en desacuerdo | 5. Algo de acuerdo | 6. De acuerdo | 7. Totalmente de acuerdo |
|---|---|---|---|---|---|---|---|
| | ◯ | ◯ | ◯ | ◯ | ◯ | ◯ | ◯ |

25. La aplicación hizo conveniente para mí comunicarme con mis pacientes. *

| | 1. Totalmente en desacuerdo | 2. En desacuerdo | 3. Algo en desacuerdo | 5. Ni de acuerdo ni en desacuerdo | 5. Algo de acuerdo | 6. De acuerdo | 7. Totalmente de acuerdo |
|---|---|---|---|---|---|---|---|
| | ◯ | ◯ | ◯ | ◯ | ◯ | ◯ | ◯ |

26. Usando la aplicación, tuve muchas más oportunidades de interactuar con mis pacientes *

| | 1. Totalmente en desacuerdo | 2. En desacuerdo | 3. Algo en desacuerdo | 5. Ni de acuerdo ni en desacuerdo | 5. Algo de acuerdo | 6. De acuerdo | 7. Totalmente de acuerdo |
|---|---|---|---|---|---|---|---|
| | ◯ | ◯ | ◯ | ◯ | ◯ | ◯ | ◯ |

27. Me sentí seguro de que cualquier información que envié a mis pacientes a través de la aplicación sería recibida *

| | 1. Totalmente en desacuerdo | 2. En desacuerdo | 3. Algo en desacuerdo | 5. Ni de acuerdo ni en desacuerdo | 5. Algo de acuerdo | 6. De acuerdo | 7. Totalmente de acuerdo |
|---|---|---|---|---|---|---|---|
| | ○ | ○ | ○ | ○ | ○ | ○ | ○ |

28. Me sentí cómodo comunicándome con mis pacientes a través de la aplicación *

| | 1. Totalmente en desacuerdo | 2. En desacuerdo | 3. Algo en desacuerdo | 5. Ni de acuerdo ni en desacuerdo | 5. Algo de acuerdo | 6. De acuerdo | 7. Totalmente de acuerdo |
|---|---|---|---|---|---|---|---|
| | ○ | ○ | ○ | ○ | ○ | ○ | ○ |

29. ¿Alguna sugerencia adicional para el mejoramiento de la App? *

This content is neither created nor endorsed by Microsoft. The data you submit will be sent to the form owner.

Microsoft Forms
